# Flowers, Ferns, and Ward Decorations

**Published:** 1886-11-13

**Authors:** 


### FLOWERS, FERNS, AND WARD DECORATIONS.

[Continued from page 10 .—Communications for this column should be addressed "Gardener," care of the Editor of THE HOSPITAL.

**My Ward my Home.**

A SISTER in a large London hospital once said to me, "My ward is my home; I am never so happy elsewhere." An outsider, to whom a hospital represents all that is gloomiest and most dismal, might think this a surprising remark, but could he behold the ward in question his opinion would undergo considerable modification, so bright and cheery it is, so well-cared for, and beautiful. My own experience of such places has taught me that in no grade of society whatsoever can one find so lively and contented a community as may be encountered within the precincts of a thriving, well-managed institution of this kind, everybody having so much to do that idleness and her satellites can find no space for the soles of their undesirable feet; and though it might occur to some people to think that the constant sight of pain and suffering would have a depressing influence on mind and spirit alike, I imagine that the consciousness, that everything possible is being done to alleviate and restore, is accountable in some measure for the light-heartedness observable throughout the whole working of the hospital.

**In a Hospital Ward.**

No pains have been spared to make the ward I have mentioned as pretty and homelike as its capabilities will admit. Pots of plants and cut flowers cover the tables and window-sills, and the former last nearly all the year round. Their presence shows clearly that some sweet things are impervious, for a time at least, to the injurious effects of antiseptics and gas. The walls are adorned with prints and pictures, mostly presented by visitors or the friends of some of those who have spent their time in the hospital service. Many may be working there still, whilst some have passe d onwards by promotion, matrimonial or otherwise, through the big iron gates into the world beyond. Most of the wards in this building are similarly decorated, some more elaborately, some considerably less. In some you will find well-filled cases of ferns and mosses and such green things as rejoice in shade and moisture; in others, aquaria full of water-plants and insects, with gold-fish and their silver relatives swimming about in the amphibicus company of green frogs and a peculiar variety of water-snails. A great deal, of course, depends on the particular hobby of the sister in charge and her staff of nurses, one lady being addicted to still life, while her next-door neighbour has a leaning in favour of something livelier; each in her own way doing her utmost to carry out her schemes satisfactorily, and, as a rule, succeeding, though not without most minute attention and care.

**London Smoke.**

Everyone knows what London smoke and dust mean in connection with plant-growing, and though there may be no climate like it, as far as humanity is concerned, the straggling, non-blooming, horticultural abortions to be seen in the metropolis tell another tale altogether. "There are two sides to every shilling," and the same species which grows like a weed in the country, and fills the garden with bloom, refuses in London to reward the utmost carefulness and coaxing. Often and often in gardening, as in other things, "virtue is its own reward," there being nothing to show for our trouble, beyond a lanky collection of limp and sickly leaves, bud and blossom being conspicuous by their absence. It is best, therefore, to grow ferns and strong hardy shrubs, the foliage of which is their chief attraction, for the decoration of London windows, and trust to the nearest nurseryman for the rest, if one would avoid cultivating blossom and disappointment at the same time.

**Decoration and Emulation.**

Ward decoration, like a good many other things, is none the worse for the accumulating effects of emulation. Where one room is adorned, and made as far as possible a thing of beauty, others in the same building will be kept up to the same mark of attractiveness; and one reason why these things are better managed than formerly lies no doubt with the different social standing of our latter-day nurses, the lady who is to-day to be found ministering to the sick and injured, being more open to refining influences than her predecessors of the Gamp and Prigg class, to whose sonorous nostrils the odour of gin was more grateful than that of violets, and vastly more comforting. Although it is the rule nowadays to find the hospital wards in London and elsewhere more or less decorated, I need not say there are exceptions, and much remains to be done throughout the kingdom. Innumerable opportunities are offered to the benevolent and wealthy for exercising their charity towards their less fortunate brethren, in the way of infirmary and workhouse decorations. Happily, there is an increasing movement on foot (though not carried half far enough) in favour of supplying the hospitals with cut flowers and other means of ornamentation. Many people, however, with the best intentions in the world, conduct their plans in so slipshod and unmethodical a fashion that partial, if not total, failure is the only recognisable result.

*(To be continued.)*

